# Cancer-Associated Fibroblast-Derived FGF7 Promotes Clear Cell Renal Cell Carcinoma Progression and Macrophage Infiltration

**DOI:** 10.3390/cells13221824

**Published:** 2024-11-05

**Authors:** Man Jia, Mingyu Xie, Xixi Luo, Huiping Wang, Chunyan Duan, Wanni Lai, Rongyang Dai, Ronghao Wang

**Affiliations:** 1 Department of Biochemistry and Molecular Biology, School of Basic Medical Sciences, Southwest Medical University, Luzhou 646000, China; 2 Department of Genetics, Xuzhou Medical University, Xuzhou 221004, China

**Keywords:** CAFs, FGF7, ccRCC

## Abstract

As the predominant stromal cells in the ccRCC surrounding environment, cancer-associated fibroblasts (CAFs) have been established as supportive of tumor growth. However, the detailed molecular mechanisms underlying the supporting role of CAFs in ccRCC have not been well characterized. Evidence from the clustering consensus analysis, single-cell analysis, and the experimental results illustrate that CAF-derived FGF7 plays a crucial role as a signaling mediator between CAFs and ccRCC tumor cells. Mechanistically, CAF-derived FGF7 triggers AKT activation to promote cell growth and cell invasion of ccRCC tumor cells. As a response, ccRCC tumor cells stimulate STAT3-mediated transcriptional regulation, directly increasing FGF7 expression at the chromatin level in CAFs. Moreover, there exists a positive clinical correlation between the abundance of CAFs, FGF7 expression, and the infiltration of M2 type macrophages. The RENCA in vivo mouse model also confirmed that FGF7 depletion could impede RCC development by reducing the recruitment of M2 type macrophages. Overall, this study delineates a key signaling axis governing the crosstalk between CAFs and ccRCC tumor cells, highlighting FGF7 as a promising therapeutic target of ccRCC.

## 1. Introduction

Clear cell renal cell carcinoma is the major type of renal cancer and poses a serious threat to a wide range of people [1]. Patients diagnosed with early-stage ccRCC exhibit a favorable prognosis, with a 5-year survival rate reaching 90%. However, approximately 25–30% of ccRCC cases progress to metastatic disease, resulting in a markedly reduced 5-year survival rate of only 20–30% and accounting for the primary cause of mortality [2,3]. In clinical practice, the mainstay treatment for localized ccRCC involves surgical removal of tumor lesions. Conversely, for patients with metastatic ccRCC, administration of tyrosine kinase inhibitors (TKIs) to suppress tumor neovascularization is the first-line therapeutic approach [4,5,6]. Sunitinib and pazopanib, approved by the U.S. Food and Drug Administration (FDA), are such inhibitors to treat metastatic ccRCC [7,8]. Several clinical trials have evidenced that TKI treatment significantly increases the progression-free survival (PFS) and overall survival (OS) of patients with metastatic ccRCC. However, TKI therapy can only extend the survival duration by approximately 5–15 months before patients develop drug resistance [9]. Therefore, identification of novel targets causally linked to ccRCC development remains a scientific topic in this field.

A tumor is a heterogeneous mass mainly consisting of cancer cells, immune cells, fibroblasts, endothelial cells, and extracellular matrix [10]. Among these, cancer-associated fibroblasts (CAFs) have a crucial impact on the tumor initiation, tumor progression, and therapy resistance in various cancers, including ccRCC [11,12,13,14]. Studies have demonstrated that CAFs could deliver exosomal miR-181d-5p to down-regulate the expression of ring finger protein 43 (RNF43), thereby facilitating RCC progression [15]. In addition, the significant infiltrated CAFs remodel an immunosuppressive microenvironment that mitigates the killing ability of cytotoxic T cells, promoting tumor growth and metastasis [16]. Nevertheless, the detailed molecular mechanisms responsible for the communication between CAFs and ccRCC cancer cells remain largely unknown, necessitating in-depth investigations.

Fibroblast growth factors (FGFs) are polypeptide growth factors with a structural resemblance, primarily functioning to promote the proliferation and differentiation of fibroblast cells via binding to the receptor proteins (FGFR) [17,18]. Mounting evidence suggests that the aberrant regulation of FGF/FGFR signaling is intricately linked to tumor progression and drug response. Early in 1991, K Fujimoto et al. [19] discovered significantly high serum levels of FGFs in RCC patients compared to healthy controls, with a strong correlation between FGF levels and RCC tumor progression [20,21], suggesting that FGFs can serve as molecular markers for RCC diagnosis and prognosis. In addition, high expression levels of FGFR1 and FGFR2 were frequently observed in RCC [21]. However, how FGFs mediated the crosstalk between ccRCC tumor cells and CAFs has not yet been investigated.

In this study, by conducting clustering consensus analysis using the biomarkers of CAFs, we found that FGF7 is highly expressed in CAF-high ccRCC patients as compared to CAF-low cohorts. The in vitro experimental evidence suggested that CAF-derived FGF7 can promote the cell growth and cell invasion of ccRCC tumor cells, which are mitigated by a specific inhibitor of the PI-3K/AKT signaling pathway. Subsequently, ccRCC tumor cells paracrinally activated STAT3 signaling in CAFs to transcriptionally regulate FGF7 expression. Additional evidence further suggested a tight correlation between CAF-derived FGF7 and macrophage infiltration in ccRCC. Notably, depletion of FGF7 could suppress ccRCC tumor growth by impeding M2 type macrophage infiltration. Overall, our study highlights the clinical significance of FGF7 in ccRCC development and provides an overarching rationale to develop FGF7-targeted therapies.

## 2. Materials and Methods

### 2.1. Bioinformatic Analyses

RNA sequencing datasets and their related clinical information were downloaded from UCSC Xena (http://xena.ucsc.edu/, accessed on 9 March 2023). Gene ontology (GO) and Kyoto Encyclopedia of Genes and Genomes (KEGGs) enrichment analyses were conducted using the “ClusterProfiler” package in R (https://github.com/YuLab-SMU/clusterProfiler, accessed on 9 March 2023). The “ConsensusClusterPlus” R package (https://github.com/renzhonglu/ConsensusClusterPlus, accessed on 9 March 2023) was used to group TCGA KIRC into CAF-high and CAF-low, which were then visualized in two dimensions by principal component analysis (PCA) and t-distributed stochastic neighbor embedding (t-SNE) analysis with “ggplot2” and “Rtsne” package, respectively. CAF-high and CAF-low ccRCC were subjected to immune cell infiltration analysis as previously described.

### 2.2. Cell Culture

Human ccRCC cell lines OSRC-2 and A498 were purchased from Xiamen Immocell Biotechnology Co., Ltd. (Xiamen, China). The mouse RENCA cell line was gifted from Dr. Lei Yin in Shanghai Jiaotong University. Cells were maintained in 10% certified FBS DMEM (VivaCell, Shanghai, China) supplemented with 2 mM L-glutamine, 100 IU/mL penicillin, and 100 µg/mL streptomycin.

### 2.3. Plasmid Construction and Lentivirus Generation

The shRNAs against mouse FGF7 were constructed into the PLKO backbone vector. To generate lentivirus, PLKO or PLKO-shFGF7 (20 µg) was co-transfected with psPAX2 packaging plasmid (10 µg) and pMD2.G envelope plasmid (10 µg) into 293T cells using the standard calcium phosphate transfection method. Lentivirus supernatant was collected using a 0.45 µm filter upon 48 h transfection and incubated with NIH3/3T cells in the presence of 5 µg/mL polybrene (TSA0101, Tsingke Biotech, Bejing, China). The infected cells were selected with 1 mg/mL puromycin (P8230, Solarbio, Beijing, China) for two weeks before the experiments.

### 2.4. Western Blotting

Cells were lysed with RIPA buffer. Protein concentration was measured with a BCA kit (ZJ101L, Epizyme Biotech, Shanghai, China), and an equal amount of protein was separated by 8–12% SDS-PAGE and transferred onto a nylon membrane (10600001, Amersham, Maidstone, UK). The membranes were blocked in 5% BSA or 5% milk for 45 min at room temperature (RT) and blotted with primary antibody overnight at 4 °C, followed by 1 h incubation of conjugated secondary antibodies, and visualized with ODYSSEY CLX (LI-COR, Lincoln, NE, USA). The primary antibodies used in this study were: FGF7 (ab131162, Abcam, Fremont, CA, USA), p-AKT-S473 (9271S, CST, Danvers, MA, USA), p-AKT-T308 (13038S, CST, Danvers, MA, USA), AKT (9272S, CST, Danvers, MA, USA), STAT3 (9139S, CST, Danvers, MA, USA), p-STAT3-Y705 (9145S, CST, Danvers, MA, USA), and β-actin (66009-1-Ig, proteintech, Rosemont, IL, USA).

### 2.5. Real-Time Quantitative PCR (RT-qPCR)

One µg total RNA extracted by TRIzol reagent (R401-01, Vazyme, Nanjing, China) was utilized for reverse transcription (RT) as the standard protocol described. Then, the RT product was diluted 20-fold and subjected to real-time quantitative PCR in a LightCycler 480 with QuantiNova SYBR Green dye (Q712-02, Vazyme, Nanjing, China). Expression of the gene of interest was normalized to the actin mRNA level. Primers used in this study are listed in Table S1.

### 2.6. Cell Proliferation Assay

RCC cells were seeded into 96-well plates at the concentration of 3000 cells/well. Cell viability was determined as OD450 value in a micro-plate reader by using the Cell-Counting Kit-8 (CCK8) assay (K101823133EF5E, APE*BIO, Houston, TX, USA) at different time points.

### 2.7. 5-Ethynyl-2′-Deoxyuridine (EdU) Assay

A BeyoClickTM EdU Cell Proliferation Kit with Alexa Fluor 555 (C0075S, Beyotime, Shanghai, China) was used according to the manufacturer’s instructions. Briefly, RCC cells with gene manipulations or various treatments were seeded into 48-well plates at the concentration of 1 × 10^4^ cells/well and incubated with 10 µM EdU for 2 h at 37 °C. Next, cells were fixed and stained with Hoechst 33342. The fluorescent images were obtained under Olympus IX73.

### 2.8. Transwell Assay

Serum-free DMEM diluted standard matrigel (356235, Corning Inc., Corning, NY, USA) was loaded into an 8 µm pore sized upper chamber and allowed to dry for 30 min. The harvested RCC cells were seeded into the upper chambers at the concentration of 5 × 10^4^ cells/well in serum-free medium. Post 16 h incubation, the invaded cells were fixed by 75% ethanol and visualized by 0.1% crystal violet (G1062, Solarbio, Beijing, China) staining. The statistical analysis of the invaded cells was performed in ImageJ software v1.8.0.345 which was downloaded with this link: https://imagej.net/imagej-wiki-static/Welcome, accessed on 19 November 2023.

### 2.9. In Vivo RCC Animal Model

A quantity of 1 × 10^6^ RENCA cells were subcutaneously co-implanted with NIH/3T3 cells (5:1) into the renal capsule of 6-week-old C57bl/6 mice. After 6 weeks, the mice were anesthetized and sacrificed for tumor weight examination and image capture. Tumors were either stored in liquid nitrogen or fixed in 4% paraformaldehyde for IHC staining.

### 2.10. Immunohistochemical Staining (IHC)

The in-house ccRCC micro-array sections comprising 62 ccRCC samples and 27 adjacent kidney tissues obtained from the pathology department of the First Affiliated Hospital of Southwest Medical University or RENCA-derived tumors were deparaffinized and subjected to antigen retrieval in citrate buffer (pH = 6.0), followed by incubation of 10% goat serum at room temperature for 1 h. Then, tissue sections were incubated with primary antibodies, including anti-CD163 (93498, CST, Danvers, MA, USA), anti-ACTA2 (ab220179, Abcam, Fremont, CA, USA), anti-FGF7 (ab131162, Abcam, Fremont, CA, USA), and Ki67 (12202, CST, Danvers, MA, USA) at 4 °C overnight, followed by signaling amplification of the second biotin-conjugated antibody and streptavidin-conjugated HRP. A DAB kit (DA1016, Solarbio, Beijing, China) was used to visualize the amplified signals.

### 2.11. Chromatin Immunoprecipitation (ChIP)

The cells were washed with 1XPBS and cross-linked using 1% formaldehyde for 15 min, followed by a 10 min incubation with 125 mM glycine. Then, the cells were lysed, and nuclear extracts were used for sonication to obtain 300–500 bp DNA fragments. After centrifugation at 4 °C, the supernatant was incubated with p-STAT3-Y705 (9145S, CST, Danvers, MA, USA) or H3K4me3 (61379, Active Motif, Shanghai, China) primary antibody overnight at 4 °C. After being pre-cleared using 5 mg/mL salmon sperm DNA and 5% BSA, protein A/G beads were utilized to precipitate DNA–protein complex. After washing 10 times with a high-salt buffer, the DNA–protein complex was digested by proteinase K, and chromatin DNAs were purified for qPCR detection.

### 2.12. Statistics

Statistical analyses were conducted by performing a one-way ANOVA test in Graphpad Prism 8.0. All the in vitro experiments were performed in triplicate and repeated three times. All values are presented as mean ± SD. * *p* < 0.05 was considered significantly different.

## 3. Results

### 3.1. Computational Identification of FGF7 as a Potential Driver During ccRCC Development

**Figure 1:**
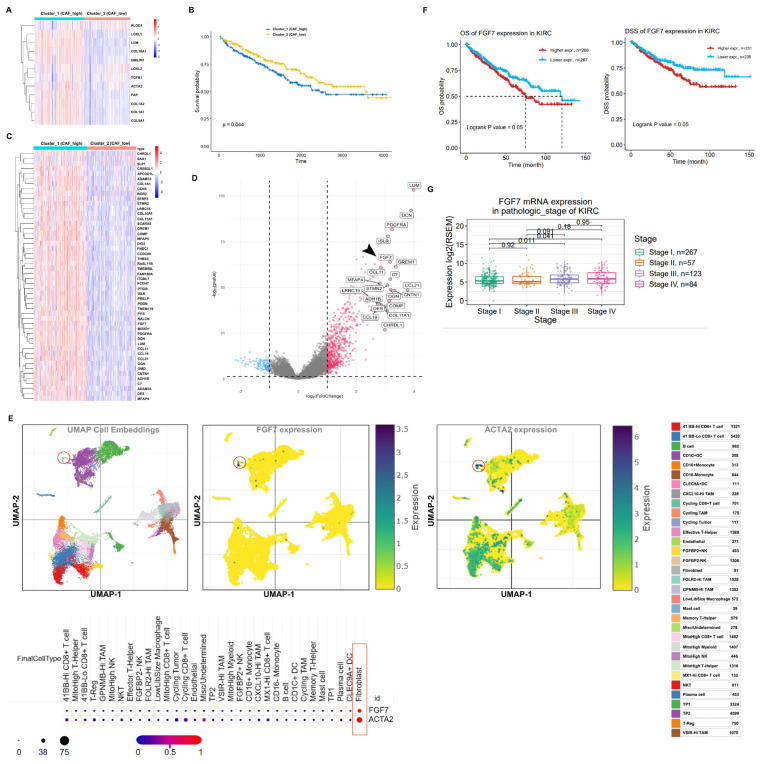
Computational identification of FGF7 as a potential driver during ccRCC development. (**A**) The heatmap of CAF biomarkers in cluster 1 and cluster 2. (**B**) CAF-high (cluster 1) ccRCC have poor OS compared to CAF-low (cluster 2) control. (**C**) The heatmap of top 50 DEGs between cluster 1 and cluster 2. (**D**) FGF7 as a significant DEG according to the volcano plot analysis. The black arrow marks the location of FGF7. (**E**) Single cell analysis of FGF7 is ccRCC (ACTA2 as positive control). CAF population is marked by the red circle and the expression levels of FGF7 and ACTA2 in fibroblast are indicated within the red box. (**F**) The ccRCC patients with high FGF7 expression have shorter OS (Left) and DSS (Right). (**G**) FGF7 expression in different pathological stages of ccRCC.

It is still intriguing to explore the molecular basis of the crosstalk between CAFs and ccRCC tumor cells. To this end, we conducted a consensus clustering analysis, categorizing ccRCC patients into CAF-high (Cluster 1) and CAF-low (Cluster 2) based on the classical CAF biomarkers (Figure 1A and Figure S1A,B). As expected, CAF-high patients experience shorter overall survival (OS) as compared to CAF-low controls (Figure 1B), strengthening the association between CAF abundance and adverse prognosis in ccRCC patients. Next, to identify the molecules closely related to the biological function of CAFs, we screened the differentially expressed genes (DEGs) between CAF-high and CAF-low groups (Figure 1C,D). The subsequent GO-KEGG analysis (Figure S1C) revealed a strong enrichment of extracellular matrix related the signaling pathway, suggesting a role for CAFs in remodeling the ccRCC environment. Among the DEGs, FGF7 garnered our attention due to its high abundance in CAFs (ACTA2 as positive control) according to the single-cell analysis of ccRCC (singlecell.broadinstitute.org) (Figure 1E). High FGF7 expression was correlated with inferior OS or disease-specific survival (DSS) (Figure 1F), with advanced ccRCC cases showing increased FGF7 expression (Figure 1G). Taken together, all these data suggest a potential role of CAF-derived FGF7 in driving ccRCC development by transmitting CAF signals to tumor cells.

### 3.2. FGF7 Promotes ccRCC Progression

**Figure 2:**
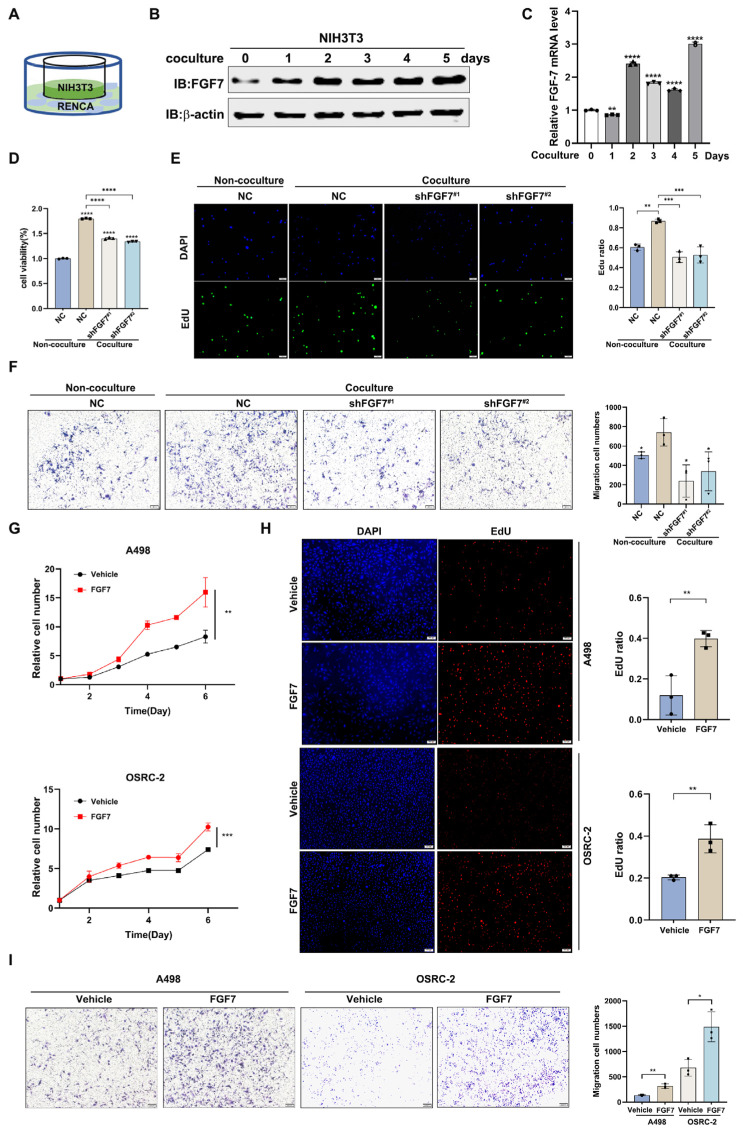
FGF7 promotes ccRCC progression. (**A**) A cartoon of co-culture. (**B**) Western blotting analysis showed that FGF7 protein level in NIH/3T3 cells was increased in the co-culture system. The b-actin was utilized as the loading control. (**C**) qPCR assay revealed that FGF7 mRNA levels in NIH/3T3 cells was increased in the co-culture system. (**D**) CCK8 assay showed that knockdown of FGF7 attenuated co-culture induced cell viability of RENCA cells. (**E**) EdU assay indicated that knockdown of FGF7-attenuated co-culture induced cell proliferation of RENCA cells. Left, representative images of EdU staining. Right, a statistical analysis. (**F**) Transwell invasion assay demonstrated that knockdown of FGF7 attenuated co-culture-induced cell invasion of RENCA cells. Left, representative images of invading cells. Right, a statistical analysis. (**G**) CCK8 assay illustrated that FGF7 treatment (20 ng/mL) accelerated the cell proliferation of both A498 (top) and OSRC-2 cells (bottom). (**H**) EdU assay showed that FGF7 (20 ng/mL) treatment promoted cell proliferation of both A498 (top) and OSRC-2 cells (bottom). Left, representative images of EdU staining. Right, the statistical analyses of EdU staining. (**I**) Transwell invasion assay demonstrated that FGF7 treatment (20 ng/mL) promoted cell invasion of both A498 and OSRC-2 cells. Left, representative images of invading cells. Right, the statistical analyses of invading cells. Scale bar = 100 μm. * *p* < 0.05, ** *p* < 0.01, *** *p* < 0.001, **** *p* < 0.0001. NC = negative control.

To examine whether CAF-derived FGF7 contributes to ccRCC development, we first transformed NIH/3T3 into CAFs by coculturing them with RENCA renal cancer cells (Figure 2A). The results revealed that CAF-related genes were drastically induced in NIH/3T3 cells upon 48 h coculture (Figure S2A), indicating a successful mimicry of CAFs in vitro. Next, we examined FGF7 mRNA and protein levels in NIH/3T3 cells before and after the coculture with RENCA cells. As shown in Figure 2B,C, the levels of both FGF7 mRNA and protein were elevated in NIH/3T3 cells upon stimulation by RENCA cells, whereas no significant changes were observed in the absence of coculture (Figure S2B,C), implying FGF7 may be functionally involved in their communication. To confirm this, we established FGF7-depleted NIH/3T3 cells (Figure S2D) and observed that FGF7 depletion could attenuate coculture-induced cell proliferation and cell invasion of RENCA cells (Figure 2D–F), monitored by CCK8/EdU and transwell invasion assay, respectively. In addition, treatment with 20 ng/mL FGF7 alone was sufficient to increase the cell proliferation, and cell invasion of OSRC-2 and A498 cells (Figure 2G–I), suggesting that the induced FGF7 expression is a prerequisite for CAFs to promote ccRCC progression. Collectively, all this evidence proves that CAF-derived FGF7 is a driver of ccRCC development.

### 3.3. FGF7 Activates AKT Signaling to Promote ccRCC Progression

**Figure 3:**
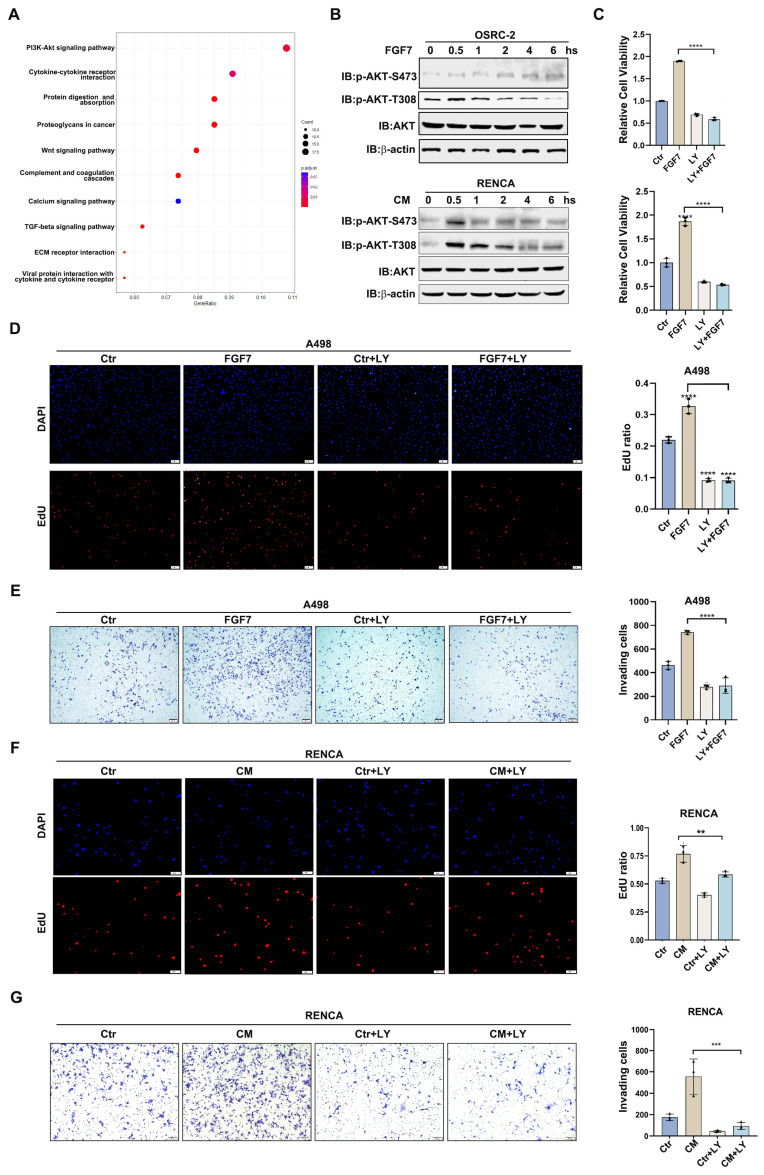
FGF7 activates AKT signaling to promote ccRCC progression. (**A**) KEGG analysis of the top 500 DEGs between FGF7-high and FGF7-low ccRCC patients. (**B**) CM and FGF7 treatment (20 ng/mL) activated renal AKT signaling, as monitored by the phosphorylation levels of AKT-S473 and AKT-T308. The β-actin was the loading control. (**C**) CCK8 assay showed that 10 μM AKT inhibitor LY294002 (LY) significantly blocked FGF7-induced cell viability of A498 (top) and OSRC-2 (bottom) cells. (**D**) EdU assay revealed that 10 μM LY dramatically attenuated FGF7-induced cell proliferation of A498. Left, representative images of EdU staining. Right, a statistical analysis. (**E**) Transwell invasion assay demonstrated that 10 μM LY treatment reversed FGF7-induced cell invasion of both A498. Left, representative images of invading cells. Right, a statistical analysis. (**F**) EdU assay revealed that 10 μM LY treatment significantly blocked CM-induced cell proliferation of RENCA cells. Left, representative images of EdU staining. Right, a statistical analysis. (**G**) Transwell invasion assay showed that 10 μM LY treatment significantly blocked CM-induced cell proliferation of RENCA cells. Left, representative images of invading cells. Right, a statistical analysis. Scale bar = 100 μm. ** *p* < 0.01, *** *p* < 0.001, **** *p* < 0.0001, n.s. = no significance.

To identify which signaling pathway is responsible for FGF7-mediated ccRCC progression, we extracted the top 500 genes highly correlated with FGF7 expression from the TCGA-KIRC dataset and conducted the GO-KEGG pathway analysis. As presented in Figure 3A, the PI-3K/AKT signaling pathway was significantly enriched and caught our attention. Consistent with this analysis, treatment of FGF7 (20 ng/mL) or coculture medium (CM) robustly activated the PI-3K/AKT signaling in renal tumor cell lines, as monitored by the phosphorylation levels of AKT at S473 and T308 (Figure 3B). To ascertain whether AKT activation is responsible for the biological effects of FGF7 or CM, we pre-treated renal cells with PI-3K/AKT signaling inhibitor (LY294002, LY) and examined whether this inhibition could block the biological responses induced by FGF7 or CM. Indeed, LY294002 exposure obviously attenuated FGF7 induced cell proliferation and cell invasion of both OSRC-2 and A498 cells (Figure 3C–E and Figure S3A,B). Consistent results were observed in the coculture system, where LY294002 treatment significantly mitigated the CM-induced cell proliferation, and cell invasion of RENCA cells (Figure 3F,G). With these findings, we conclude that CAF-derived FGF7 triggers PI-3K-/AKT activation to promote ccRCC development.

### 3.4. STAT3 Transcriptionally Regulates FGF7 Expression in CAFs

**Figure 4:**
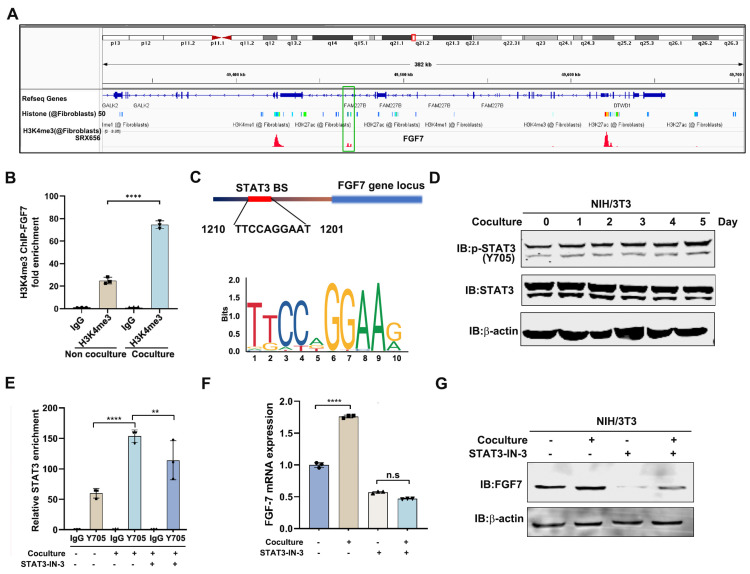
STAT3 transcriptionally regulates FGF7 expression in CAFs. (**A**) H3K4me3 peak analysis of FGF7 gene locus in fibroblasts according to the ChIP-seq Peak dataset (*ChIP-Atlas: Peak Browser*). The FGF7 gene locus at the chromosome is marked by the red box and the H3K4me3 peak in the promoter region of FGF7 gene locus is marked by the green box. (**B**) H3K4me3 ChIP-qPCR revealed that co-culture promoted H3K4me3 enrichment in the promoter region of FGF7. (**C**) JASPAR software predicted a conserved STAT3 binding sequence in the promoter region of FGF7. (**D**) Western blotting analysis of STAT3-Y705 level in NIH/3T3 cells before and after the co-culture with RENCA cells. The b-actin served as internal control. (**E**) STAT3-Y705 ChIP-qPCR revealed that co-culture increased the STAT3 enrichment in the promoter region of FGF7, which was attenuated by 5 μM STAT3-IN-11 treatment. (**F**) A qPCR assay revealed that 5 μM STAT3-IN-11 treatment reduced co-culture-induced FGF7 mRNA levels in NIH/3T3 cells. (**G**) Western blotting analysis showed that 5 μM STAT3-IN-11 blocked co-culture-induced FGF7 protein expression in NIH/3T3 cells. The β-actin was used as loading control. ** *p* < 0.01, **** *p* < 0.0001, n.s. = no significance.

We were prompted to explore the molecular mechanism underlying the increased FGF7 expression in CAFs. We hypothesized that FGF7 is regulated at the chromatin level owing to the increased FGF7 transcript in NIH/3T3 cells post-coculture. To elucidate this, we analyzed the promoter activity signature, represented by H3K4me3 peak, of FGF7 (within the gene locus of FAM227B) in fibroblasts (Figure 4A), and examined its enrichment in our coculture system. As illustrated in Figure 4B, the H3K4me3 levels in the promoter region of FGF7 gene locus were notably enriched in RENCA-educated NIH/3T3 cells, suggesting FGF7 is under transcriptional regulation in this context. To identify the transcription factor controlling FGF7 expression, we utilized JASPAR software (http://jaspar.elixir.no) to screen the promoter region of FGF7 and found it contains a conserved binding site of STAT3 (Figure 4C). In addition, Western blotting analysis demonstrated that STAT3 in NIH/3T3 was activated by CM in a time-dependent manner, as monitored by the phosphorylation level of STAT3-Y705 (Figure 4D). The subsequent ChIP-qPCR also consolidated the binding of STAT3 to the promoter region of FGF7, with increased enrichment in the coculture system (Figure 4E). Consistently, coculture-induced STAT3 enrichment on the promoter region of FGF7 was abolished by a specific STAT3 inhibitor STAT3-IN-11 (Figure 4E). Significantly, STAT3-IN-11 treatment could block RENCA-induced FGF7 expression in NIH/3T3 cells (Figure 4F,G). Collectively, all these data substantiate the notion that STAT3 is a key transcription factor controlling FGF7 expression in CAFs cells.

### 3.5. FGF7 Has a Close Relationship with M2 Macrophage Infiltration

**Figure 5:**
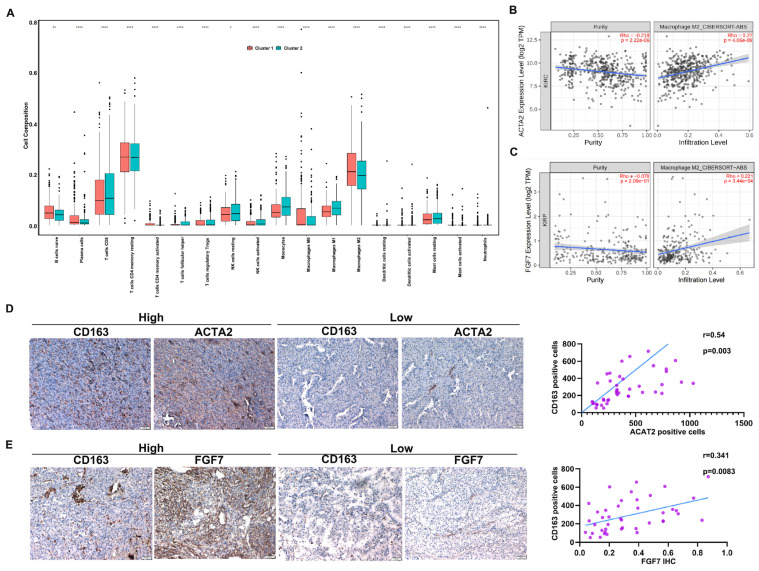
FGF7 has a close relationship with M2 macrophage infiltration. (**A**) The infiltration of immune cells between CAF-high and CAF-low ccRCC patients. (**B**,**C**) TIMER analyses showed that both ACTA2 (**B**) and FGF7 expression (**C**) were positively correlated with the infiltration level of M2 type macrophages. (**D**) ACTA2 has a positive correlation with CD163+ macrophage population. Left, representative images. Right, a statistical analysis. (**E**) FGF7 expression had a positive correlation with CD163+ macrophage population. Left, representative images. Right, a statistical analysis. Scale bar = 100 μm. * *p* < 0.05, ** *p* < 0.01, **** *p* < 0.0001.

The infiltration of immune cells within a tumor mass plays various roles during tumor development. We performed an immune cell infiltration analysis to explore the association between CAF signature and the infiltration of immune cells. As the data exhibited in Figure S4A,B and Figure 5A show, T cells and macrophages abundantly exist in the ccRCC surrounding environment, and a differential infiltration of immune cells was observed between the CAF-high and CAF-low groups. Particularly, the macrophage population (M1, M2) has a close correlation with the CAF signature. M2 type macrophages prefer to be infiltrated in CAF-high (cluster1) ccRCC tumors while M1 type macrophages selectively occupy CAF-low tumors, implying CAFs have a tight association with the recruitment of M2 type macrophages, which may establish an immune suppressive environment to support ccRCC tumor growth. In addition, analyses from TIMER suggested that the expression level of ACTA2, as well as FGF7, is positively correlated with the M2 macrophage population (Figure 5B,C), which was confirmed by IHC staining of CD163, ACTA2, and FGF7 in our established ccRCC tissue micro-array (Figure 5D,E). Taken together, all this evidence suggests that CAFs derived FGF7 has a close association with the infiltration of M2 type macrophages, which may support ccRCC development.

### 3.6. Depletion of FGF7 in CAFs Hinders ccRCC Growth

**Figure 6:**
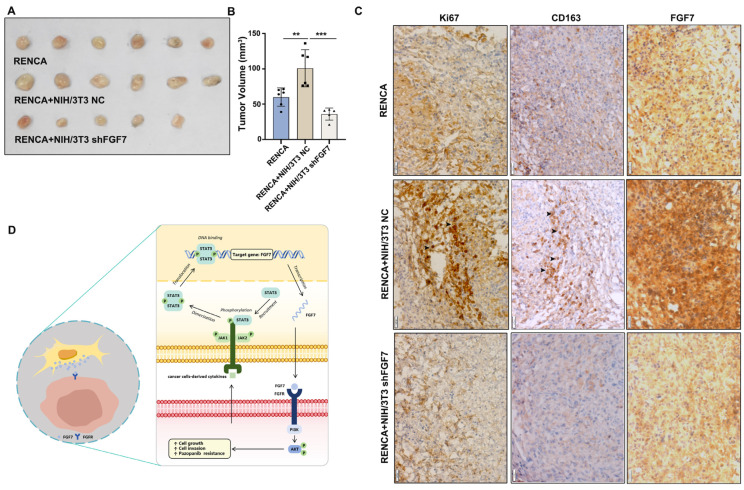
Depletion of FGF7 in CAFs hinders ccRCC growth. (**A**) Representative images of RENCA tumors. (**B**) The tumor volume of RENCA tumors. (**C**) IHC staining of Ki67, CD163, and FGF7 in RENCA tumors. (**D**) The working model of this study. Scale bar = 100 μm. ** *p* < 0.01, *** *p* < 0.001. NC = negative control.

We were prompted to observe the in vivo efficacy of targeting the FGF7 in ccRCC model. To do this, we subcutaneously implanted RENCA cells with indicated treatments: (1) without NIH/3T3 cells; (2) with control NIH/3T3 cells (at 5:1 ratio); (3) with shFGF7 NIH/3T3 cells (at 5:1 ratio) into 6-week-old male C57bl/6 mice and monitored tumor growth. As shown in Figure 6A,B, RENCA cells with NIH/3T3 grew bigger tumors than the RENCA cells alone. Notably, FGF7 depletion blocked NIH/3T3-induced RENCA tumor growth, suggesting the tumor-promoting role of FGF7 in CAFs in vivo. Similarly, RENCA-NIH/3T3 tumors exhibited higher levels of Ki67 as compared to RENCA tumors (Figure 6C), which were attenuated upon FGF7 knockdown in NIH/3T3. As previously mentioned, CAF-high ccRCC patients suffer a high infiltration of macrophages and an immune-suppressive micro-environment. Consistently, a marked increase in M2 macrophage infiltration was observed in RENCA-NIH/3T3 tumors as compared to the controls, which was mitigated by FGF7 depletion (Figure 6C).

Together, our study demonstrates a signaling axis involving STAT3/FGF7/AKT that mediates the crosstalk between CAFs and ccRCC tumor cells (Figure 6D), providing valuable insights for treating ccRCC patients.

## 4. Discussion

A tumor is a complicated ecosystem containing a wide range of non-cancerous cells, which greatly influence its progression and therapeutic effectiveness. Increasing evidence suggests that the CAF population plays a crucial role in ccRCC evolution. In this study, we computationally and experimentally identified CAF-derived FGF7 as a causal factor driving ccRCC tumor growth via at least activating the PI-3K/AKT signaling pathway. Reciprocally, tumor cells induced STAT3 signaling to up-regulate FGF7 expression at the transcript level in CAFs. In addition, the in vivo ccRCC mouse model also substantiated the tumor-promoting role of CAF-derived FGF7, as its depletion significantly hindered tumor growth and the recruitment of M2 type macrophages. Overall, our study clearly configures a potential targeted signaling axis linking CAFs and ccRCC cancer cells, providing a rationale to develop FGF7-targeted therapy against ccRCC progression.

### 4.1. CAF-Derived FGF7 Contributes to PI-3K/AKT/mTOR Activation in ccRCC

Hyper-activation of PI-3K/AKT signaling as well as its downstream target mTORC1 has been frequently observed in a subset of ccRCC patients [22], leading to the commencement of a clinical trial investigating everolimus therapy on ccRCC [23]. PI-3K/AKT/mTORC1 signaling is initiated by PtdIns-3,4,5-P3-mediated PDK1 recruitment, which subsequently phosphorylates AKT at T308 [24]. Additionally, AKT is a substrate of mTORC2, which directly phosphorylates AKT at S473 [25]. Our evidence demonstrated an elevation in the phosphorylation levels of T308 and S473 upon FGF7 or CM treatment, suggesting both PDK1 and mTORC2 were involved in FGF7-induced AKT activation. Indeed, FGFR1b/FGFR2b, as the specific FGF7 receptor, has been reported as the upstream tyrosine kinase receptor (RTK) enabling activation of the PI-3K/AKT and mTORC2/AKT pathways. The sustained up-regulation of FGF7 in CAFs represents a key factor accounting for the clinical hyper-activation of AKT as well as mTORC1 in ccRCC. Therefore, blocking FGF7 signaling emerges as a promising clinical strategy to inhibit AKT activation and mitigate ccRCC development.

### 4.2. STAT3 Is a Novel Transcript Factor for FGF7 Expression in CAFs

In an early study in 2023, Chen et al. demonstrated that FGF7 overexpression and STAT3 activation are simultaneously examined in nicotine- and silica-induced pulmonary fibrosis [26]. However, the direct linkage between STAT3 and FGF7 has not yet been explored. In this study, we found that there is a very conserved STAT3 binding site in the promoter region of FGF7, indicating STAT3 may serve as the upstream TF regulating FGF7 transcription. Subsequent experimental results confirmed the recruitment of STAT3 to the promoter region of FGF7, controlling its expression. We noticed that STAT3-induced FGF7 expression in CAFs still occurs even in the absence of tumor cells because STAT3 inhibition by STAT3-IN-11 is sufficient to decrease FGF7 expression (Figure 4G), suggesting a regulation of FGF7 expression by the basal activity of STAT3 in CAFs. The communication between CAFs and tumor cells is largely fulfilled by the extracellular molecules. As a good responder, STAT3 has been documented to regulate a bundle of gene expression after the external stimulation. This study implements that STAT3 could also regulate FGF7 expression in CAFs to drive ccRCC development. Of note, we only utilized the 2kb promoter region of FGF7 to predict the potential TFs. It is gradually being recognized that TFs can regulate gene expression via binding the distant enhancer region. Therefore, it is largely possible that FGF7 expression in CAFs can be regulated by other TFs, and continuous investigation should be made to configure this regulation.

### 4.3. Targeting FGF7 Signaling May Overcome ccRCC Progression

Owing to the critical roles of FGF proteins in various disease models, FGFR-targeted therapies have been clinically investigated. For instance, FGF401, a selective FGFR4 inhibitor is currently undergoing a phase I/II trial for the treatment of hepatocarcinoma [27,28]. AZD4547, an inhibitor of FGFR1-3, is also being clinically tested in several tumors [29,30]. FGF7 subfamily proteins, including FGF3, FGF7, FGF10, and FGF22, preferably bind FGFR1b/FGFR2b to exert biological functions [31]. Theoretically, inhibition of FGFR1/FGFR2 with small molecules could potentially attenuate FGF7-induced ccRCC progression. Infigratinib, as an FGFR1/2/3 inhibitor, has been approved to treat locally advanced or metastatic cholangiocarcinoma [32] and could be a candidate for ccRCC therapy. Moreover, lirafugratinib, a potent and highly selective FGFR2 inhibitor, is currently undergoing clinical trials for treatment of metastatic cholangiocarcinoma or other malignant solid tumors [33,34], holding promise as a therapeutic option for ccRCC.

## Data Availability

The original contributions presented in the study are included in the article/Supplementary Materials, further inquiries can be directed to the corresponding authors.

## Supplementary Materials

The following supporting information can be downloaded at: https://www.mdpi.com/article/10.3390/cells13221824/s1, Figure S1: A, The Consensus matrix. B, the PCA analysis of CAF_high and CAF_low. C, The GO-KEGG analysis of the DEGs between CAF_high and CAF_low; Figure S2: A, qPCR assay showed that co-culture increased the expression levels of CAFs related genes in NIH/3T3 cells. B, Western blotting showed FGF7 expression has no obvious changes in NIH/3T3 without RENCA treatment. The b-actin was used as loading control. C, qPCR assay demonstrated that FGF7 expression has no obvious changes in NIH/3T3 without RENCA treatment. D, Western blotting showed that the knockdown efficiency of shRNAs against FGF7 in NIH/3T3. The b-actin was used as loading control; Figure S3: A, EdU assay revealed that 10 mM LY treatment dramatically attenuated FGF7 induced cell proliferation of OSRC-2 cells. Left, representative images of EdU staining. Right, a statistical analysis. B, The transwell invasion assay demonstrated that 10 mM LY treatment reversed FGF7 induced cell invasion of OSRC-2 cells. Left, representative images of invading cells. Right, a statistical analysis; Figure S4: A, The estimated proportion of immune cells in cluster 1 (CAF_high). B, The estimated proportion of immune cells in cluster 2 (CAF_low); Table S1: The list of primers used in quantitative real time PCR.
