# Supplementary material for: Cancer-Associated Fibroblast-Derived FGF7 Promotes Clear Cell Renal Cell Carcinoma Progression and Macrophage Infiltration
Source: Cells. 2024 Nov 5;13(22):1824. doi: 10.3390/cells13221824 (PMC11593278; doi:10.3390/cells13221824)
Supplement: Supplementary file 1 [file cells-13-01824-s001.zip › supp figure legends.pdf]

Supporting information

Supplementary Figure legend

Figure S1. A, *The Consensus matrix.* ***B,*** *the PCA analysis of CAF_high and CAF_low.* ***C,*** *The GO-KEGG analysis of the DEGs between CAF_high and CAF_low.*

Figure S2. A*, qPCR assay showed that co-culture increased the expression levels of CAFs related genes in NIH/3T3 cells.* ***B,*** *Western blotting showed FGF7 expression has no obvious changes in NIH/3T3 without RENCA treatment. The b-actin was used as loading control.* ***C****, qPCR assay demonstrated that FGF7 expression has no obvious changes in NIH/3T3 without RENCA treatment.* ***D,*** *Western blotting showed that the knockdown efficiency of shRNAs against FGF7 in NIH/3T3. The b-actin was used as loading control.**p<0.05, **p<0.01, ***p<0.001, ****p<0.0001.*

Figure S3*.* ***A,*** *EdU assay revealed that 10 mM LY treatment dramatically attenuated FGF7 induced cell proliferation of OSRC-2 cells. Left, representative images of EdU staining. Right, a statistical analysis.* ***B****, The transwell invasion assay demonstrated that 10 mM LY treatment reversed FGF7 induced cell invasion of OSRC-2 cells. Left, representative images of invading cells. Right, a statistical analysis. Scale bar=100 mm. *p<0.05, **p<0.01, ****p<0.0001.*

Figure S4. A*, The estimated proportion of immune cells in cluster 1 (CAF_high).* ***B****, The estimated proportion of immune cells in cluster 2 (CAF_low).*

Table S1. The list of primers used in quantitative real time PCR.
