# Supplementary material for: Cancer-Associated Fibroblast-Derived FGF7 Promotes Clear Cell Renal Cell Carcinoma Progression and Macrophage Infiltration
Source: Cells. 2024 Nov 5;13(22):1824. doi: 10.3390/cells13221824 (PMC11593278; doi:10.3390/cells13221824)
Supplement: Supplementary file 1 [file cells-13-01824-s001.zip › Table S1.pdf]

Table S1. The primers used for qPCR or ChIP

Name	Sequences (5'→3')
COL5A1	Forward Primer: CTTCGCCGCTACTCCTGTTC<br>Reverse Primer: CCCTGAGGGCAAATTGTGAAAA
COL16A1	Forward Primer: TGGGACAAATATAGGTGAGCGG<br>Reverse Primer: CGTTGGTTGATGGGTCACTACT
COL1A2	Forward Primer: TCGTGCCTAGCAACATGCC<br>Reverse Primer: TTTGTCAGAATACTGAGCAGCAA
COL1A1	Forward Primer: GCTCCTCTTAGGGGCCACT<br>Reverse Primer: ATTGGGGACCCTTAGGCCAT
LOXL1	Forward Primer: GAGTGCTATTGCGCTTCCC<br>Reverse Primer: GGTTGCCGAAGTCACAGGT
LUM	Forward Primer: CTCTTGCCTTGGCATTAGTCG<br>Reverse Primer: GGTCATCACAGTACATGGCAGT
PLOD3	Forward Primer: ATGTGGCTCGAACAGTTGGTG<br>Reverse Primer: TTGCCAGAATCACGTCGTAGC
Beta-actin	Forward Primer: GGCTGTATTCCCCTCCATCG<br>Reverse Primer: CCAGTTGGTAACAATGCCATGT
mFGF7	Forward Primer: ACCTGAGGATTGACAAACGAGG<br>Reverse Primer: CCACGGTCCTGATTTCCATGA
mFGF7-ChIP	Forward Primer: ACTTACCAACCTCCTCCGTG<br>Reverse Primer: AGACCTCTGATTCCCAAGCC
