# Supplementary figures and images for: Cancer-Associated Fibroblast-Derived FGF7 Promotes Clear Cell Renal Cell Carcinoma Progression and Macrophage Infiltration
Source: Cells. 2024 Nov 5;13(22):1824. doi: 10.3390/cells13221824 (PMC11593278; doi:10.3390/cells13221824)

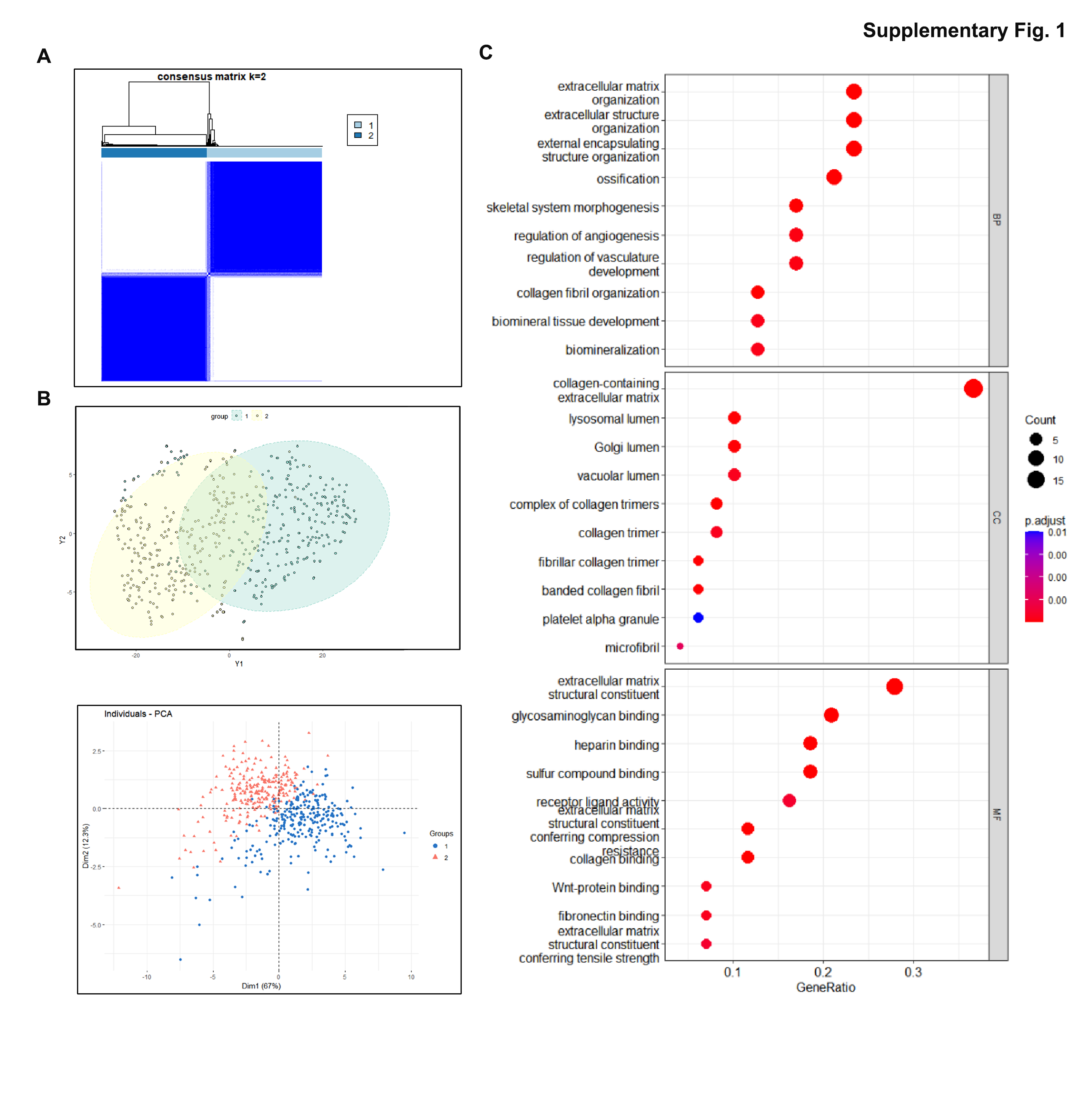

Supplement: Supplementary file 1 [file cells-13-01824-s001.zip › Figure S1.tif]

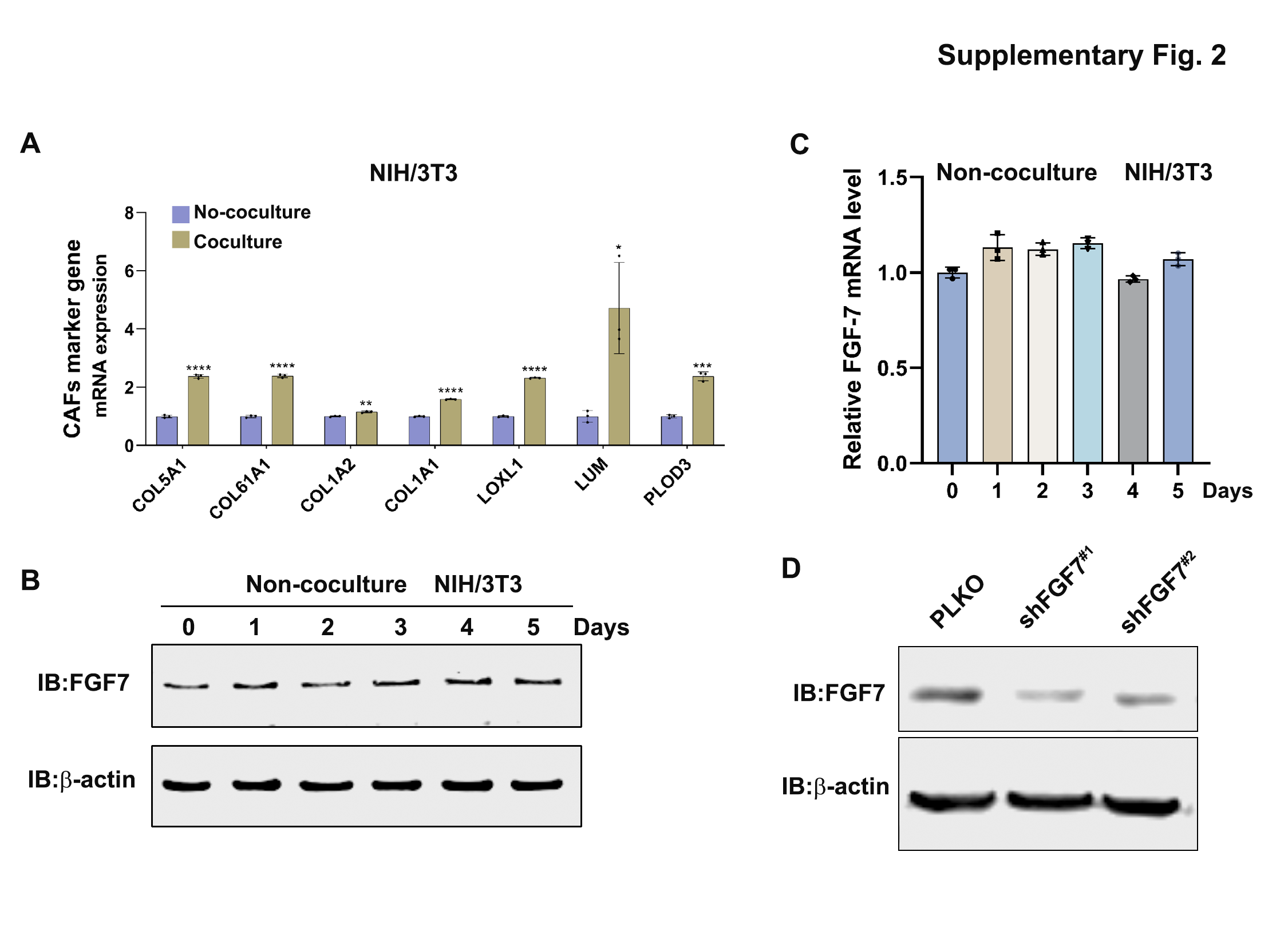

Supplement: Supplementary file 1 [file cells-13-01824-s001.zip › Figure S2.tif]

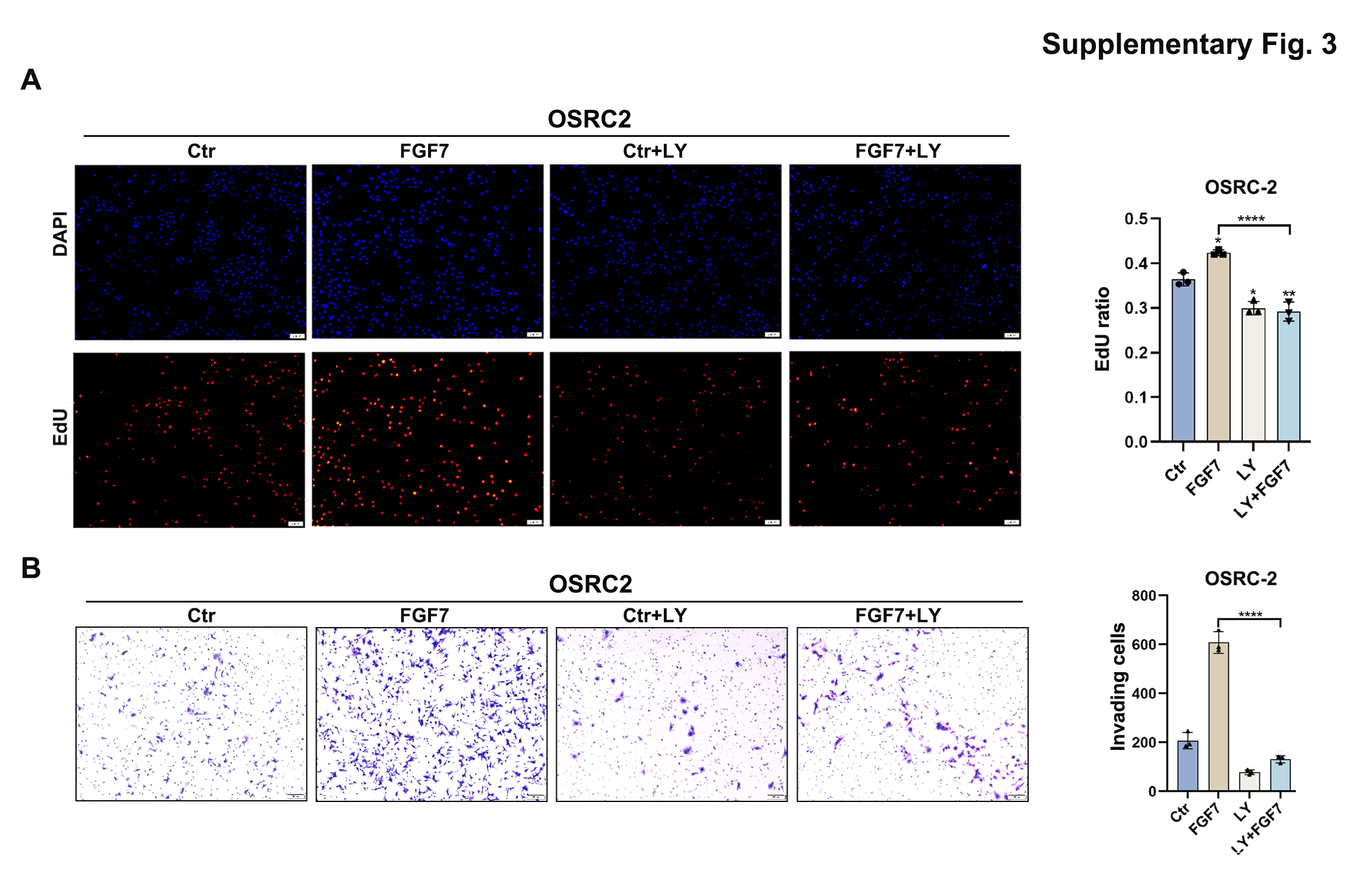

Supplement: Supplementary file 1 [file cells-13-01824-s001.zip › Figure S3.tif]

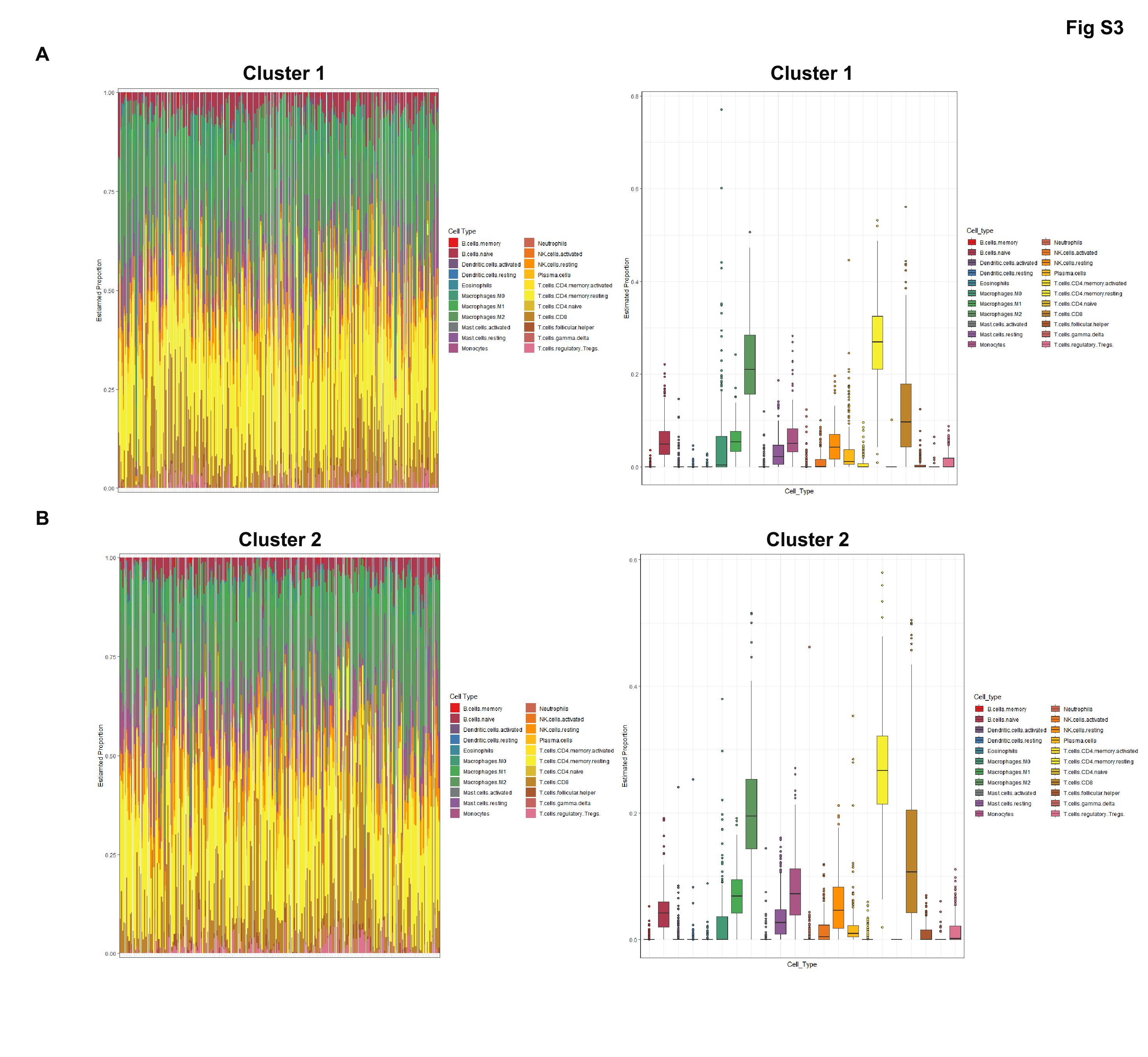

Supplement: Supplementary file 1 [file cells-13-01824-s001.zip › Figure S4.tif]
